# Comparison of Composition and Anticaries Effect of *Galla Chinensis* Extracts with Different Isolation Methods

**DOI:** 10.2174/1874210601711010447

**Published:** 2017-08-31

**Authors:** Xuelian Huang, Meng Deng, Mingdong Liu, Lei Cheng, R.A.M. Exterkate, Jiyao Li, Xuedong Zhou, Jacob. M. Ten Cate

**Affiliations:** 1 State Key Laboratory of Oral Diseases, West China Hospital of Stomatology, Sichuan University, Chengdu, China; 2 Department of Cariology, Endodontology, Pedodontology, Academic Centre for Dentistry Amsterdam (ACTA), Amsterdam, The Netherlands; 3 Division of General Dentistry, Eastman Institute for Oral Health, University of Rochester, Rochester, NY, USA; 4 Oral Biology Program, School of Dentistry, University of North Carolina, Chapel Hill, NC, USA; 5 Department of Analytical Toxicology, West China School of Preclinical and Forensic Medicine, Sichuan University, Chengdu, China

**Keywords:** *Galla chinensis*, Gallic acid, Composition, Dental caries, Cariogenic microbiota

## Abstract

**Objectives:**

*Galla chinensis* water extract (GCE) has been demonstrated to inhibit dental caries by favorably shifting the demineralization/remineralization balance of enamel and inhibiting the biomass and acid formation of dental biofilm. The present study focused on the comparison of composition and anticaries effect of *Galla chinensis* extracts with different isolation methods, aiming to improve the efficacy of caries prevention.

**Methods:**

The composition of water extract (GCE), ethanol extract (eGCE) and commercial tannic acid was compared. High performance liquid chromatography coupled to electrospray ionization-time of flight-mass spectrometry (HPLC-ESI-TOF-MS) analysis was used to analyze the main ingredients. *In vitro* pH-cycling regime and polymicrobial biofilms model were used to assess the ability of different *Galla chinensis* extracts to inhibit enamel demineralization, acid formation and biofilm formation.

**Results:**

All the GCE, eGCE and tannic acid contained a high level of total phenolics. HPLC-ESI-TOF-MS analysis showed that the main ingredients of GCE were gallic acid (GA), while eGCE mainly contained 4-7 galloylglucopyranoses (GGs) and tannic acid mainly contained 5-10 GGs. Furthermore, eGCE and tannic acid showed a better effect on inhibiting enamel demineralization, acid formation and biofilm formation compared to GCE.

**Conclusions:**

*Galla chinensis* extracts with higher tannin content were suggested to have higher potential to prevent dental caries.

## 1 INTRODUCTION

Dental caries, the most prevalent and costly oral infectious disease worldwide, is attributed to prolonged plaque acidification resulting from the metabolic activity of cariogenic microbiota, which leads to demineralization of the tooth [1-3]. Although fluoride’s cariostatic efficacy has been demonstrated, additional approaches are needed to enhance its effectiveness [4, 5]. Natural products have been used as a major source of innovative and effective therapeutic agents throughout human history, offering a diverse range of structurally distinctive bioactive molecules [6]. It is estimated that plants produce a vast array of more than 100,000 metabolites, and the total number of metabolites in plants exceeds 500,000 [7]. These structurally diverse substances with a wide range of biological activities could be useful for the development of alternative or adjunctive anticaries therapies. However, identification of the bioactive component or compound from thousands of chemicals still remains a fundamental challenge to develop natural product derived anticaries agents.

In recent years, one of the natural products, *Galla chinensis* extract (GCE) was demonstrated with potentials to inhibit the activities of cariogenic pathogens and favorably shift the demineralization/remineralization balance of enamel and dentine [8-12]. However, complete chemical characterization of GCE is still needed before further application. In addition, different extraction methods might influence its compositions and subsequent bioactivities. During previous studies, we noticed that, in inhibiting enamel demineralization, GCE was more effective than GCE-A, GCE-B, GCE-C and GCE-D, which were fractionated from GCE by adsorption chromatography [13]. Whereas other studies showed that the aqueous extract of *Galla chinensis* could inhibit periodontopathogenic bacteria and an ethanol extract was reported having strong antibacterial activity against *Staphylococcus aureus* [14, 15]. Tian’s studies on consecutive extracts from *Galla chinensis* showed that the extracts with weaker polarity (such as ethyl acetate and ethanol) contained gallotannins with higher molecular weight, and had stronger antioxidant and antibacterial activities [16]. Given the complex chemical nature of natural products, it still remains a challenge to screen bioactive component and optimize the isolation methods, which may finally yield the best anti-caries effect.

Therefore, the purpose of this study was to chemically characterize three *Galla chinensis* extracts: aqueous extract (GCE), ethanol extract (eGCE) and tannic acid (gallotannin, extracted from *Galla chinensis*) and to compare their anticaries effects, and identify the most effective *Galla chinensis* extract as an anticaries agent.

## 2 MATERIALS AND METHODS

### 2.1 Plant Material

*Galla chinensis* (origin: Sichuan province, China) was purchased from China Tong Ren Tang drugstore (Chengdu, China) and authenticated by Prof. Jing Huang, in the Institute of Naturally Occurring Drugs of Sichuan University, Chengdu, China.

### 2.2 Extraction of *Galla Chinensis*

*Galla chinensis* (2 kg) was dried in an oven at 60 °C for 3 days, and then finely powdered. The powder was equally divided into two portions. One was added to 600 mL of distilled water and stirred for 10 h at 65°C and then filtered. The extract was re-extracted with distilled water under the same conditions. Then the extract was dissolved in 500 mL of ethanol (95%). After filtration and evaporation, the remaining extract was lyophilized to give a powder (*Galla chinensis* aqueous extract, GCE). Another portion was added to ten times ethanol (95%). The mixture was stirred at 50 °C and then filtered. The extract was re-extracted with ethanol under the same conditions. After filtration and evaporation of the ethanol, the remaining extract was lyophilized to give a powder (*Galla chinensis* ethanol extract, eGCE). Tannic acid was purchased from Sigma (Guarantee analysis, Sigma-Aldrich, USA), which is extracted from *Galla chinensis* and is also called gallotannin.

### 2.3 Determination of Total Phenolics

Total phenolics (TP) were determined by Folin-Ciocalteu (FC) assay and expressed as gallic acid equivalents (GAE) based on a gallic acid (GA) calibration curve. FC assay was performed according to Singleton and Rossi [17], which has been modified to determine the total phenolics of *Galla chinensis* extracts previously [18]. Each sample was performed in triplicate.

### 2.4 Determination of Tannin Content

The tannin content was determined using the method described by Tian [18] and was also determined as GAE. The tannin content was the difference of total phenolics of test sample before and after precipitating casein referring to the FC assay. Each sample was performed in quadruplicate.

### 2.5 High Performance Liquid Chromatography Coupled to Electrospray Ionization-Time of Flight-Mass Spectrometry Analysis

GCE, eGCE and tannic acid were analyzed by high performance liquid chromatography coupled to electrospray ionization-time of flight-mass spectrometry (HPLC-ESI-TOF-MS) [19], a tandem system. The Agilent 1200 series HPLC consisted of a model G1315D diode array detector (DAD), a G1312B binary Pump, a G1379B vacuum degasser, a G1367C auto-sampler and a G1316B column heater. The concentration of the testing solution was 1mg/mL, and the injection volume was 5 μL. Gradient elution HPLC was applied at a flow rate of 0.4 mL/min with detection at 270 nm. Two solvents were used for the mobile phase: (A) 0.1% formic acid and 10 mM ammonium formate (pH3.0), (B) acetonitrile. Compounds were separated using the following gradient: 0-5 min, 5% B; 5-8 min, 20% B; 20-30 min, 30% B; 30-40 min, 20% B; 40.0-40.1 min, 5% B. The separation of components in GCE was performed on an Agilent column (Poroshell 120 SB-C18, 150mm × 4.6mm, particle size 2.7μm) at 40 °C.

The time of flight (TOF) mass detector (G1969A, Agilent, US) was equipped with electrospray ionization (ESI) interface. The ESI voltage was 3.5 kV, and a mass range of 50-3000 m/z was scanned in negative full scan mode. Data processing was performed on Agilent Mass Hunter (v.B.01.04) software.

### 2.6 Determination of GA Content in GCE

HPLC-DAD was equipped and the gradient elution HPLC conditions were the same as HPLC-ESI-TOF-MS analysis. The flow rate was 0.4 mL/min, and detection wavelength was 270 nm. The injection volume was 10 μL. Data processing was performed on CHEMSTATION (v.B.04.02) software. GA (reference substance) in all the studies was purchased from The National Institute for the Control of Pharmaceutical and Biological Products, Chengdu, China.

### 2.7 Effects on Demineralization of Bovine Enamel

Bovine enamel specimens were prepared and the baseline surface microhardness (SMH) was measured as in previous studies [20]. Then, forty specimens were selected from a batch of 420 specimens, with baseline SMH between 388.7 and 436.1 Knoop hardness number (KHN).

The specimens were randomly divided into the following 5 treatment groups (each N=10): 4.0 mg/mL GCE solution, 4.0 mg/mL eGCE solution, 4.0 mg/mL tannic acid solution, 1 mg/mL NaF solution (positive control), deionized distilled water (DDW, negative control). Next, specimens were pH-cycled following the procedure described in a previous study [2]. In brief, after immersion of the specimens in one of the treatment solutions for 5 min, the blocks were placed in an acidic buffer (50 mmol/L acetic acid, 1.5 mmol/L potassium dihydrogen orthophosphate, pH 5.0) for 1h (2 mL per block), and then in a neutral buffer (20 mmol/L HEPES, 1.5 mmol/L potassium dihydrogen orthophosphate, pH 7.0) for 5 min (2 mL per block) in each of 12 cycles. After each step, the specimens were washed in DDW (1 min) for 3 times.

After pH-cycling, the acidic buffers were retained for analysis. Calcium concentration in the acidic solutions was determined using atomic absorption spectrophotometry (AAS, Thermo Element MKII-M6, Thermo Electron, USA). Then a calcium depletion rate (CDR) for each sample was calculated. This is a measure of calcium extracted per unit area per unit time in the 2 mL acid and is calculated as

CDR=mass of calcium extracted(time in acid)(area exposed)(μg/(hmm2))

In addition, representative specimens from all treatment groups were randomly selected for scanning electron microscopic (SEM). Air-dried samples were sputtered with gold resulting in a gold coating. Then, the morphology of enamel surfaces was evaluated with a SEM (S450, Hitachi, Japan).

### 2.8 Effects on Polymicrobial Biofilms

Polymicrobial biofilms were formed in a high-throughput active attachment biofilm model as described in detail in previous studies and previously used to determine the effects of GCE on biofilms [21, 22]. In brief, glass disks were attached to a custom made lid, in such a way that each disk fitted into one of the wells of a polystyrene, 24-well flat-bottomed microtiter plate. Each well was filled with 1.5 ml McBain medium [23], supplemented with 0.2% (w/v) sucrose and buffered at pH 7.0 with 50 mM PIPES buffer, to submerge the disks. A saliva-glycerol stock was added (1:50 final dilution) as inoculum and the microtiter plates were incubated anaerobically at 37 °C for a total period of 48h, with refreshments of the medium after 8, 24 and 32 h. Subsequently, the disks were immersed into one of the following eight treatment solutions (4 mg/mL GCE solution, 4 mg/mL eGCE solution, 4 mg/mL tannic acid solution or DDW) for 1 h. After treatment, biofilms were harvested in PBS solution. Treatments effects were assessed from colony forming unit (CFU) and lactic acid formation data. For this, saliva had been donated by a single donor. The model was approved by the institutional review board and the donor had given written informed consent.

### 2.9 Statistical Analysis

Statistical evaluations were performed using the SPSS 16.0 software (SPSS Incorporated, Chicago). The normal distribution of the data was tested with the Shapiro-Wilk tests. T-test and One-way ANOVA was used. The level of significance was set at 5%.

## 3 RESULT

### 3.1 Determination of Total Phenolics and Tannin Content

The total phenolics and tannin content is shown in Fig. (**1**). The three *Galla chinensis* extracts had similar values in total phenolics but had different tannin content. eGCE and tannic acid had more tannin than GCE, indicating these two extracts had stronger capability to precipitate proteins.

### 3.2 HPLC-ESI-TOF-MS Analysis

HPLC-DAD chromatograms demonstrated that GCE consists of several components (Fig. **3A3B3C**), which was also revealed by total ion current chromatography (Fig. **2A & B** & **C**). In detail, the main molecular mass ranges, corresponding compounds and m/z values are shown in Table (**1**) [16, 24-26]. The m/z values revealed that GCE were mixtures, consisting mainly of GA and its isomer, and 1-3 galloylglucopyranoses (GGs), while eGCE mainly contained GA and 4-7 GGs, and tannic acid mainly contained 5-10 GGs.

### 3.3 Determination of GA Content in GCE

GA content is 71.3±0.2%, 2.8±0.02% and 0.5±0.003% (w/w) in GCE, eGCE and tannic acid, respectively, indicating that GCE contained a high amount of GA, while eGCE and tannic acid had little.

### 3.4 Effects on Demineralization of Bovine Enamel

The CDRs analysis is presented in Fig. (**4**). CDRs were significantly lower in *Galla chinensis* extracts groups and NaF group than in DDW group (ANOVA, *P* < 0.05). However, CDRs were significantly higher in GCE group than in eGCE group, tannic acid, and NaF group (ANOVA, *P* < 0.05). No significant differences of CDRs were observed among eGCE, tannic acid and NaF groups (ANOVA, *P* > 0.05). The data show that all *Galla chinensis* extracts could inhibit enamel demineralization, and GCE was less effective than eGCE and tannic acid.

**Fig. (5):**
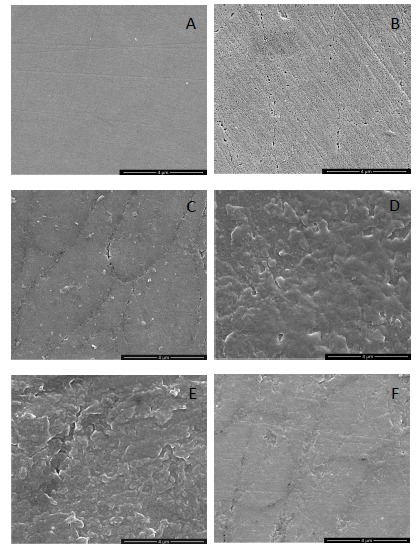
**A**. SEM images of the sound enamel (30 000×); **B**. SEM images of the enamel surfaces in DDW group (30 000×); **C**. SEM images of the enamel surfaces in NaF group (30 000×);**D**. SEM images of the enamel surfaces in GCE group (30 000×); **E**. SEM images of the enamel surfaces in eGCE group (30 000×);**F**. SEM images of the enamel surfaces in tannic acid group (30 000×).

The images of sound enamel surface (Fig. **5A**) showed an intact appearance. In contrast, the DDW treated enamel (Fig. **5B**) was disorganized, with obvious demineralization in and around enamel rod. In fluoride group (Fig. **5C**), the surface was relatively intact except demineralization around enamel rod. The surfaces of GCE, eGCE and tannic acid groups (Figs. **5D**, **E** and **F**) were similar with layers of deposition with disorganized arrangement.

### 3.5 Effects on Polymicrobial Biofilm

The lactic acid formation and the CFU values are shown in Figs. (**6A** and **6B**), respectively. GCE, eGCE and tannic acid could significantly reduce the acid production and CFU counting compared with DDW group (ANOVA, *P* < 0.05), whereas eGCE and tannic acid were more effective than GCE (ANOVA, *P* < 0.05). The data showed eGCE and tannic acid could inhibit the acid formation and biofilm formation in polymicrobial biofilms.

## 4 DISCUSSION

In the present study, the composition and anticaries effects of *Galla chinensis* extracts with different isolation methods was compared. All the GCE, eGCE and tannic acid contained high level of total phenolics, while eGCE and Tannic acid had more tannin than GCE, indicating these two extracts had stronger capability to precipitate proteins. HPLC-ESI-TOF-MS analysis further showed that the main ingredients of GCE were GA, while eGCE mainly contained 4-7 GGs and tannic acid, a commercialized gallotannin from *Galla chinensis,* mainly contained 5-10 GGs. In addition, when assessed by using *in vitro* pH-cycling regime and polymicrobial biofilm model, eGCE and tannic acid showed better effect on inhibiting enamel demineralization, acid formation and biofilm formation compared to GCE. Combined with those finding, *Galla chinensis* extracts with higher tannin content were suggested to have higher potential to prevent dental caries.

Natural products have been used as an extremely diverse source for developing innovative anti-caries reagent; however, the great complexity of biological samples still remains a major obstacle to the identification of the true effective composition. *Galla chinensis* is rich in heterogenous gallotannins and also contains nearly 20% GA, 7% methyl gallate, and some resin, fat, wax, starch, *etc* [16, 27]. Previous studies have already proved the *in vitro* efficacy of GCE as an anti-caries reagent. However, little is known about the chemical composition of GCE and its relationship with functional activities, as different extract conditions, *e.g.*, extraction solvent, temperature, pH, extraction time, particle size and other supplementary means such as microwave, would influence the yields and products [16, 28]. In the present study, we only changed two factors, extraction solvent and temperature, and found that the composition of extracts was significantly changed. GCE extracted by water at 65°C, contained less tannin content than eGCE extracted by ethanol at 50°C. This is in agreement with a previous study that gallotannins in *Galla chinensis* had weak polarity and were more soluble in non-polar or weak-polar solvents, such as ethanol compared to polar solvents, such as water [16].

As *Galla chinensis* mainly contained hydrolysable tannins, to further elucidate the detailed constituents of GCE, HPLC-ESI-TOF-MS was applied. HPLC is one of the predominant methods used to separate constituent. HPLC with gradient elution has a continuous change in the mobile phase during separation, which is essential for improving the sharpness of the peaks [29]. HPLC coupled with mass detection was widely used to identify unknown polyphenolics in biological samples [30]. The online coupling of HPLC with MS using ESI can provide efficient resolution and identification of a wide range of polar compounds, while TOF-MS can provide accurate mass data over a wide dynamic range and thereby permits the rapid and reliable assessment of the elemental composition of ions [31]. In the combined detection system, major constituents and degree of polymerization of *Galla chinensis* extract were revealed. This lays an initial basis to exploit the structure-activity relationship to develop reliable anti-caries reagent with defined chemical composition.

Inhibiting enamel demineralization is an effective strategy to prevent dental caries [32]. The process of enamel demineralization involves the dissolution of enamel apatite crystals and the diffusion of ions. It has been suggested that the diffusion pathway in enamel is controlled by the organic matrix network, which occupies both the inter and intra-prismatic spaces [33], and the changes on enamel organic matrix can affect the process of the enamel demineralization through the control of the diffusion pathway in tooth structures [34]. In a previous study, GA, the main component in GCE, has been revealed to inhibit enamel demineralization [35] and GCE-organic matrix interaction has been further found to play a substantial role in inhibiting the demineralization [36]. The enamel organic matrix consists of short peptide fragments, rich in Pro, Glx, and Gly, which are presumably breakdown products of the major enamel matrix protein, amelogenin [37]. GCE could interact with enamel organic matrix through polyphenols organic matrix interaction involving covalent, ionic, hydrogen bonding or hydrophobic processes [38, 39]. This interaction could stabilize the enamel organic matrix during the acid attack and the induced metamorphic enamel organic matrix is precipitated in the enamel and blocks the diffusion pathway of the ions in tooth structures, thus inhibiting the mineral loss from enamel [39]. In the current study, the three extracts could all inhibit enamel demineralization but showed different activities. eGCE and tannic acid were more effective than GCE in inhibiting the enamel demineralization as HPLC-ESI-TOF-MS analysis revealed that they contain more high molecular weight gallotannins. As it is reported for 1-10GGs, a higher degree of galloylation results in stronger protein binding ability, it is reasonable that eGCE and tannic acid could interact with enamel organic matrix more potently and therefore inhibit enamel demineralization more effectively compared to GCE.

The biofilms model in the present study is an active attachment rather than a sedimentation biofilm model, and saliva was used as inoculum to form polymicrobial biofilm. Thus, the model can provide new and relevant insights into the properties and dynamics of the dental plaque ecosystem [8, 21]. The resistance to killing by antimicrobial agents in biofilms increases compared with the resistance exhibited by planktonic bacteria, during which, biofilm matrix plays an important role, either by acting as a diffusion barrier, or by binding directly to antimicrobial agents and preventing their access to the biofilm cells [40]. Therefore, the interaction with target proteins in our biofilms was much more difficult, and the effects on caries pathogens in biofilms were much less [41]. In the present model, all the *Galla chinensis* extracts showed inhibitive effect on the microbial metabolism and polymicrobial biofilm formation, and eGCE (mainly with 4-7GGs) and tannic acid (mainly with 5-10GGs) have stronger inhibition effect than GCE, which were consistent with the findings that the extracts with weaker polarity contained gallotannins with higher molecular weight, and had stronger antibacterial activities [18]. It is possible that polyphenols in *Galla chinensis* extracts can interact with the membrane proteins of bacteria by means of hydrogen bonding through their hydroxyl groups, which can result in changes in membrane permeability and cause cell destruction and inhibit cell proliferation [42]. In addition, polyphenols may also penetrate into bacterial cells, disrupt the proton motive force, electron flow, active transport and coagulate cell contents, resulting in lowered lactic acid production. Besides, eGCE mainly contained 1-7GGs. 6GGs and 7GGs were demonstrated with more remarkable antibacterial activities than other gallotannins [18]. We speculate the medium molecular weight gallotannins are easier to penetrate the diffusion barrier in biofilm than high molecular weight gallotannins and are easier to react with target protein than GA and high molecular weight gallotannins, although this requires further investigation.

## CONCLUSION

Taking information on compositions and anticaries effects of three *Galla chinensis* extracts together, eGCE and tannic acid are suggested to be the most effective *Galla chinensis* extracts as anticaries agent. Further we conclude that medium molecular weight gallotannins are the most active constituent in terms of caries prevention. Optimum extraction methods should be chosen to maximize their levels in caries preventive extracts.

## Figures and Tables

**Fig. (1):**
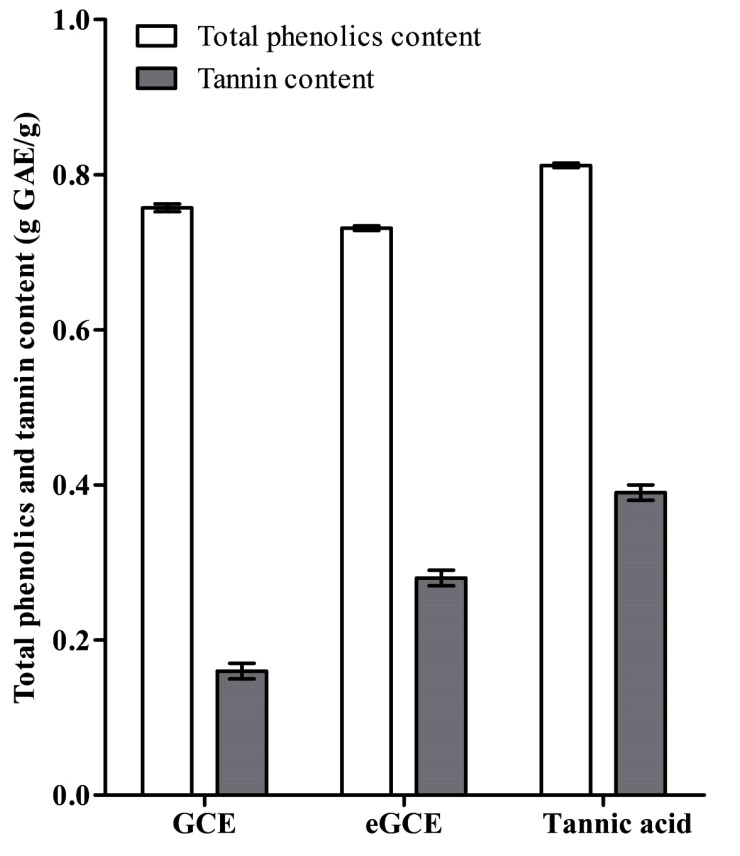
Total phenolics (n=3) and tannin content (n=4) of GCE, eGCE and tannic acid.

**Fig. (2A):**
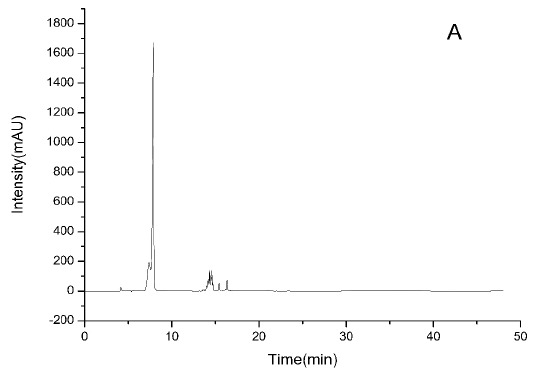
The HPLC-DAD chromatograms of GCE at 270 nm.

**Fig. (2B):**
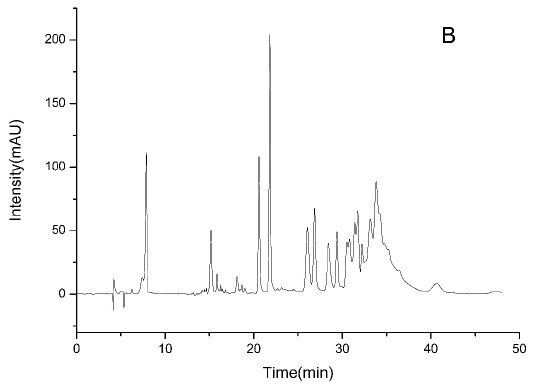
The HPLC-DAD chromatograms of eGCE at 270 nm.

**Fig. (2C):**
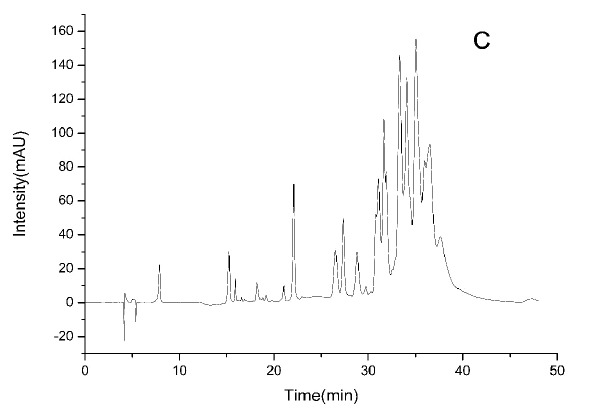
The HPLC-DAD chromatograms of tannic acid at 270 nm.

**Fig. (3A):**
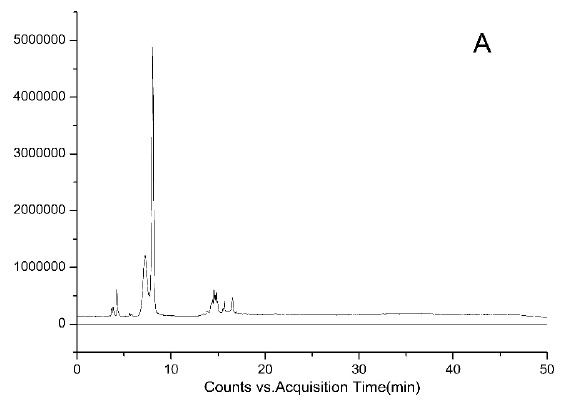
Total ion current chromatography of GCE obtained from HPLC-ESI-TOF-MS.

**Fig. (3B):**
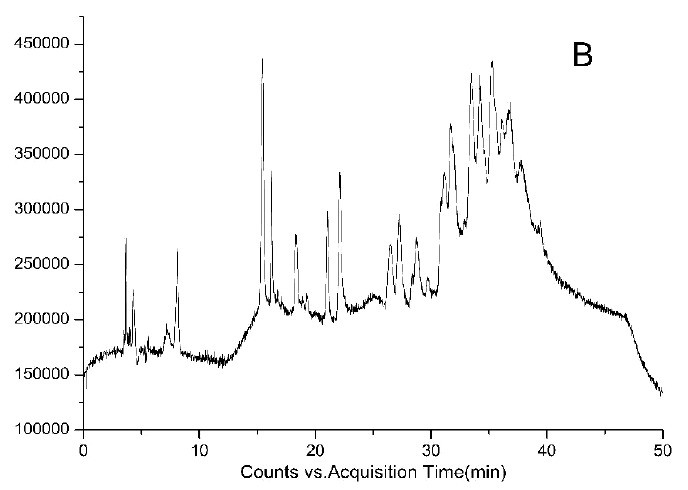
Total ion current chromatography of eGCE obtained from HPLC-ESI-TOF-MS.

**Fig. (3C):**
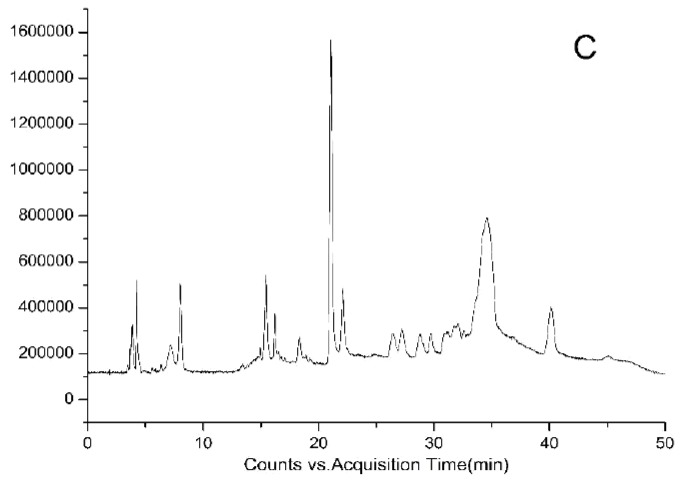
Total ion current chromatography of tannic acid obtained from HPLC-ESI-TOF-MS.

**Fig. (4):**
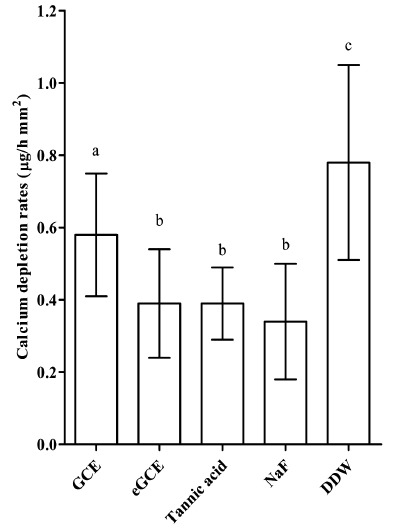
Calcium depletion rates (CDR) in acidic buffers after pH-cycling regimen. In each group, n=10. Different letters (a-c) denote statistically significant differences among groups (*P* < 0.05, ANOVA).

**Fig. (6A):**
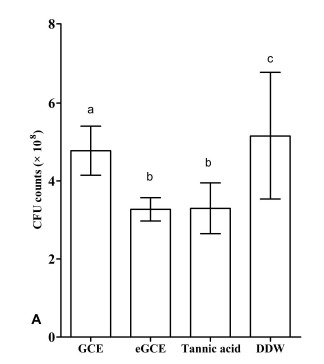
CFU counts after 48h of microcosm biofilm formation on glass substratum and treated at 48 h for 1 h with 4 mg/mL GCE solution, 4 mg/mL eGCE solution, 4 mg/mL tannic acid solution or DDW, respectively (n = 4). Different letters (a-c) denote statistically significant differences among groups (*P* < 0.05, ANOVA).

**Fig. (6B):**
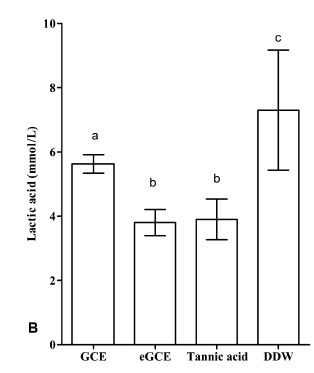
Lactic acid formation after 48 h of microcosm biofilm formation on glass substratum, and treated at 48 h for 1 h with 4 mg/mL GCE solution, 4 mg/mL eGCE solution, 4 mg/mL tannic acid solution or DDW, respectively (n = 4). Different letters (a-c) denote statistically significant differences among groups (*P* < 0.05, ANOVA).

**Table 1:** The main molecular mass range, corresponding compounds, and m/z values observed from negative ion experiments with HPLC-TOF-MS for eGCE and GCE and tannic acid.

Extracts	Main molecular mass range [M-H]^-^	Corresponding Compounds^a^	Main m/z values^b^
GCE	169- 635	GA, 1-3GGs	169,331, 483, 635
eGCE	169-1243	GA, 4-7GGs	169, 787, 939, 1091, 1243
Tannic acid	939-1699	5-10GGs	939, 1091, 1243, 1395, 1547, 1699

a 1-10GGs represented galloylglucopyranose, di-galloylglucopyranose, tri-galloylglucopyranose, tetra-galloylglucopyranose, penta-galloylglucopyranose, hexa-galloylglucopyranose, hepta-galloylglucopyranose, octa-galloylglucopyranose, ennea-galloylglucopyranose, deca-galloylglucopyranose, respectively [16, 24-26]. b 169, 331, 483, 635, 787, 939, 1091, 1243, 1395, 1547, and 1699 would match with the [M-H]- ions of gallic acid, 1-10GGs, respectively.
